# Supplementary material for: The Circulating Nucleic Acid Characteristics of Non-Metastatic Soft Tissue Sarcoma Patients
Source: Int J Mol Sci. 2020 Jun 24;21(12):4483. doi: 10.3390/ijms21124483 (PMC7349923; doi:10.3390/ijms21124483)
Supplement: Supplementary file 1 [file ijms-21-04483-s001.zip › Supplementary files/Supplementary figures IJMS final revision.docx]

The circulating nucleic acid characteristics of Non-Metastatic Soft Tissue Sarcoma patients

**Supplementary Figures**


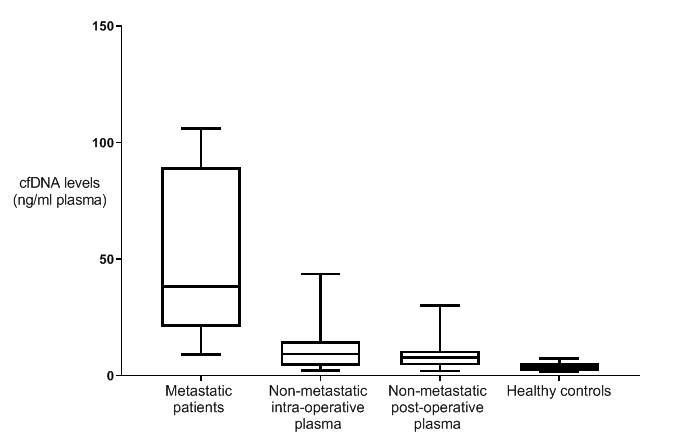


**Figure S1: Box and whisker plot showing cfDNA levels in metastatic STS patients, non-metastatic STS patients and healthy controls.** Boxes represent 25th-75th percentile, whiskers the range and horizontal lines the median. The metastatic patient and healthy control data has previously been published by our group. No significant difference was seen between the non-metastatic patient intra-operative or post-operative cfDNA levels, and healthy control cfDNA levels (p= 0.07, unpaired t-test). Metastatic patient cfDNA levels are significantly higher than cfDNA levels in every other group (p<0.0001).


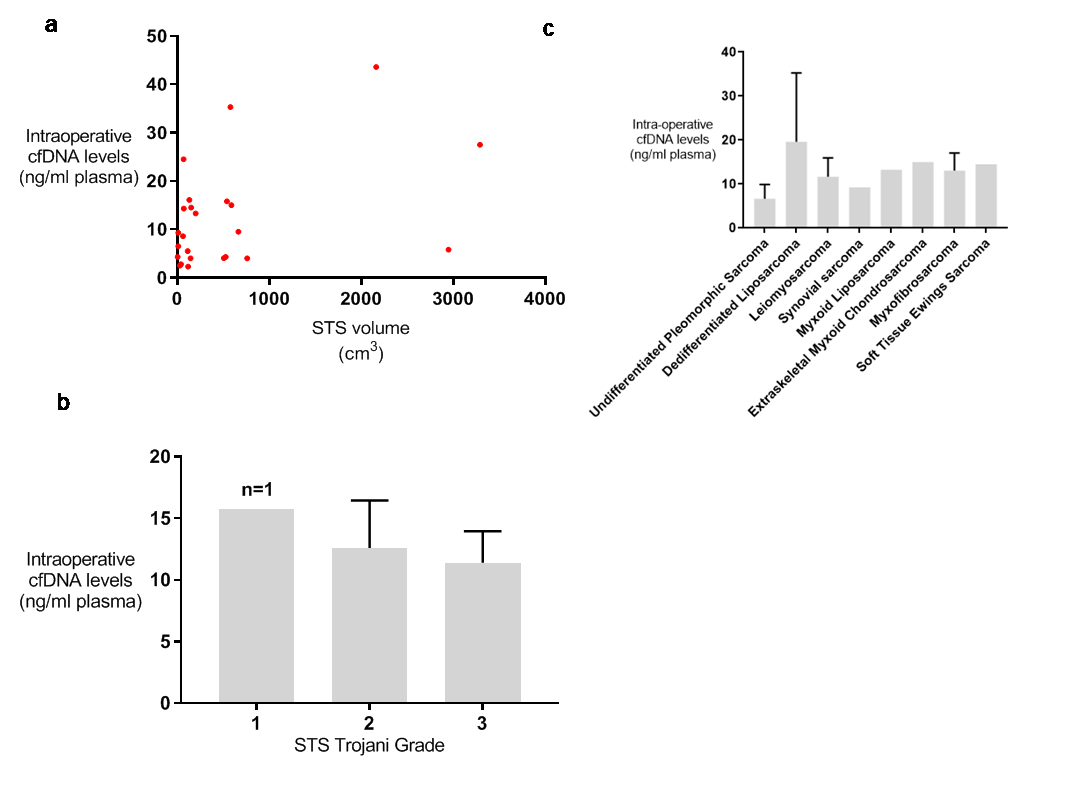


**Figure S2: Relationship between tumour characteristics and intra-operative cfDNA levels.**

(a) Comparison of STS volume and intra-operative cfDNA levels. No significant correlation was identified (R^2^=0.19).

(b) Comparison of the intra-operative cfDNA levels with tumours of different Trojani grades. The mean and standard error of the mean are represented by error bars. The mean intra-operative cfDNA level was 12.6 (±3.9 SEM, n=12) for the grade 2 tumours and 11.4 (±2.6 SEM, n=11) for the grade 3 tumours. The single grade 1 STS analysed had an intra-operative cfDNA level of 15.8ng/ml. No significant difference was seen between groups (p=0.9, ANOVA).

(c) Intra-operative cfDNA levels categorised by STS subtype. Error bars representing the SEM are shown where multiple patients with the same STS subtype were analysed (undifferentiated pleomorphic sarcoma n=4, dedifferentiated liposarcoma n=2, leiomyosarcoma n=5, myxofibrosarcoma n=10). Cell free DNA levels were not significantly different between groups (p=0.96, ANOVA).


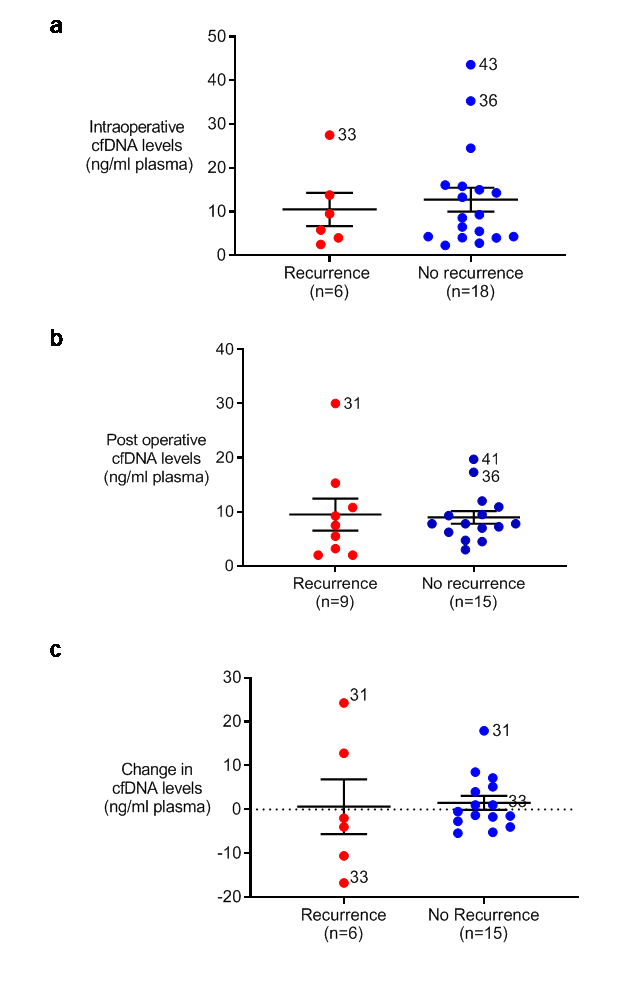


**Figure S3: Comparison of peri-operative cfDNA levels and recurrence in STS patients.** (a) Comparison of intra-operative cfDNA levels in patients that developed recurrence with patients that remained disease free during follow up. Horizontal bars represent the mean and SEM. The participant numbers of outlying patients are also shown. Mean intraoperative cfDNA levels for patients with recurrence was 10.5ng/ml (± 3.8 SEM, median 7.7, range 2.5-27.5) compared to 12.8ng/ml (± 2.7 SEM, median 9.0, range 2.3-43.6) in patients that were disease free during follow up (p=0.67, unpaired t-test.)

(b) comparison of cfDNA levels at first post-operative follow up appointments in patients with and without recurrence. Mean cfDNA levels at this stage were 9.5ng/ml (± 1.2 SEM, median 7.5, range 2-30) in patients that suffered recurrence and 9.0 ng/ml (± 1.2 SEM, median 7.8, range 3-19.7) in patients without recurrence (p=0.85, unpaired t-test).

(c) Comparison of the difference in intra- and post-operative cfDNA levels between the two groups. On average cfDNA levels rose by a mean of 0.63ng/ml (± 6.2 SEM, median -3, range -16.8-24.3) following surgery in patients that suffered recurrence. Cell free DNA rose by a mean of -1.5ng/ml (± 1.6 SEM, median -0.2, range -18-5.4) in patients that remained disease free (p= 0.85, unpaired t-test). One patient was lost to follow up.


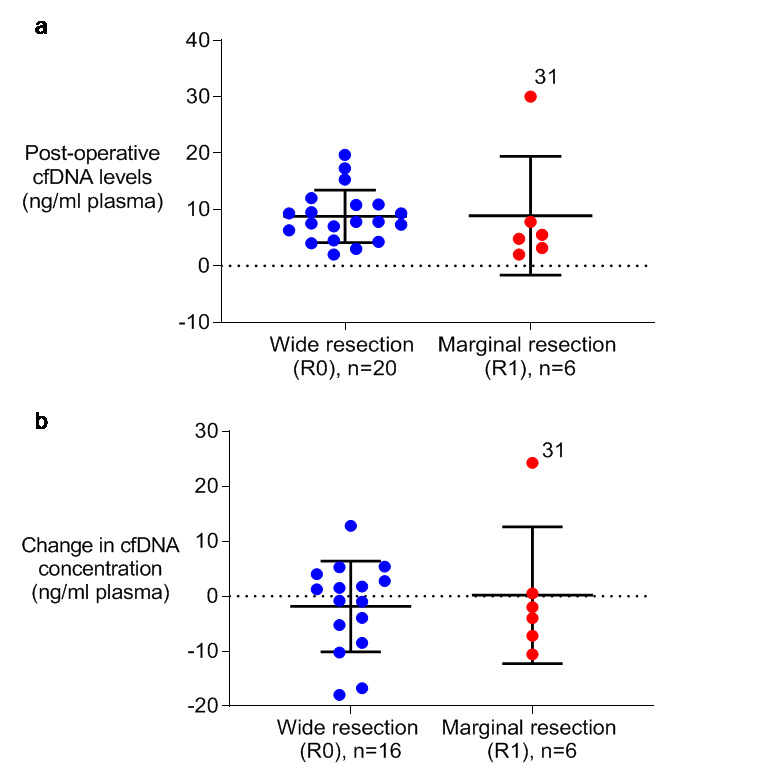


**Figure S4: Comparison of peri-operative cfDNA levels and surgical resection margins in STS patients.**

(a) Comparison of post-operative cfDNA levels in patients that underwent wide (R0) resections and marginal (R1) resections (unplanned and planned). No significant difference was seen in the post-operative cfDNA levels of these groups (p=0.97, unpaired t-test). (b) plots the difference between each patient’s intra- and post-operative cfDNA level. The graph compares these differences in patients that underwent wide or marginal resections. No significant difference was seen between groups (p= 0.61, paired t-test). The participant number of 31 is shown as potential outlier.


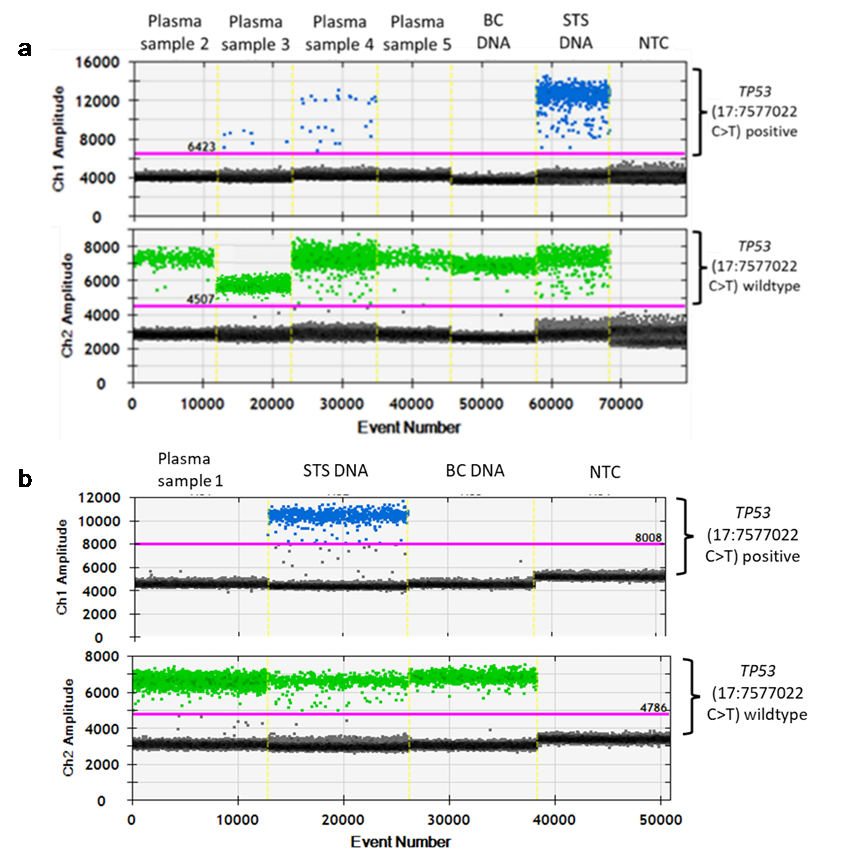


**Figure S5: Droplet digital PCR mutation analysis of patient 6 targeting *TP53* (17:7577022 C>T) / *TP53*;R306*****.**

Cumulative droplet count (event number, X axes) and the fluorescent amplitudes of channel 1 (FAM/blue=mutant allele) and channel 2 (HEX/green=wildtype allele), Y axes. (a) shows the analysis of plasma collected 4, 11, 29 and 33 weeks post-operatively with BC DNA, STS DNA and a non-template control (NTC). (b) shows the analysis of plasma collected intra-operatively with similar controls. Circulating *TP53*; R306* was identified in samples 3 (VAF 0.42%, 254 copies/ml), and 4 (VAF 1.75% (SEM 0.15), 3580 copies/ml (SEM 307)). SEM = Standard error of mean.


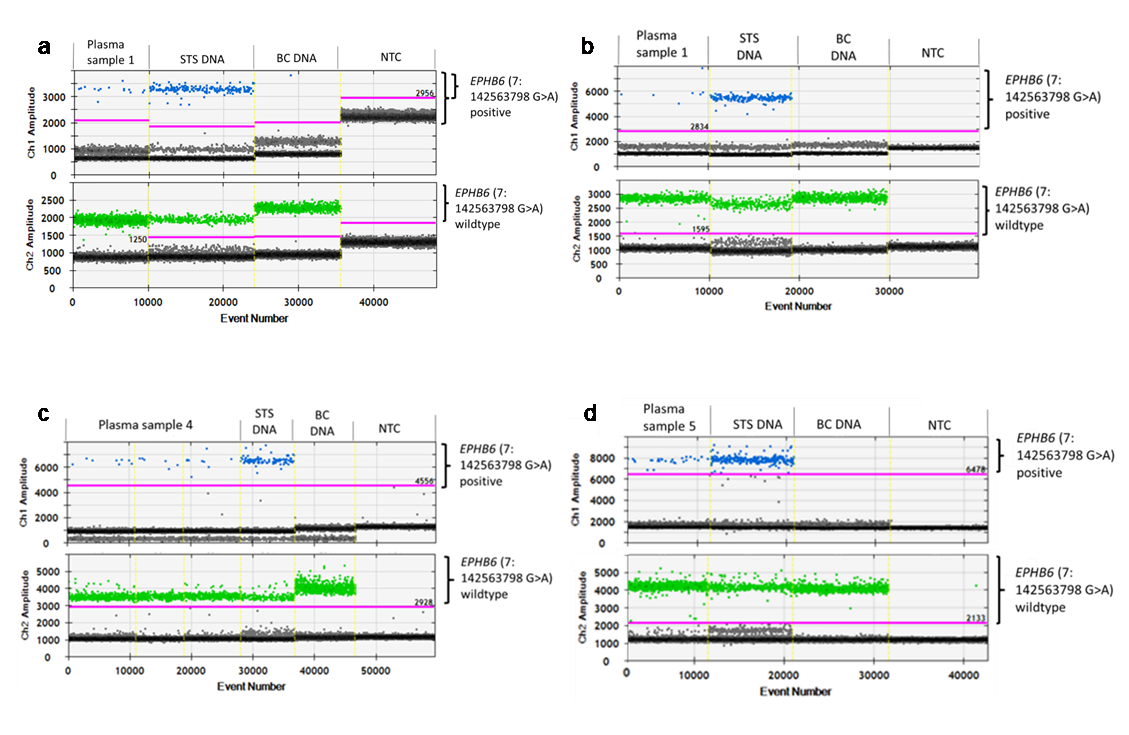


**Figure S6: Droplet digital PCR mutation analysis of patient 22 targeting *EPHB6* (7: 142563798 G>A)/ *EPHB6*;G397R.**

Cumulative droplet count (event number, X axes) and the fluorescent amplitudes of channel 1 (FAM/blue=mutant allele) and channel 2 (HEX/green=wildtype allele), Y axes. Patient 22’s BC DNA, STS DNA, and a non-template control (NTC) reaction were used as controls. Panels a/b, c and d show the analyses of plasma samples 1, 4 and 5 respectively. Circulating *EPHB6*;G397R was identified intra-operatively (VAF 0.85, SEM 0.15), in plasma sample 4 (VAF 1.6 (SEM 0.12), 7919 copies/ml (SEM 582)) and plasma sample 5 (VAF 2.1 (SEM 0.1). SEM = Standard error of mean.
