# Supplementary material for: The Circulating Nucleic Acid Characteristics of Non-Metastatic Soft Tissue Sarcoma Patients
Source: Int J Mol Sci. 2020 Jun 24;21(12):4483. doi: 10.3390/ijms21124483 (PMC7349923; doi:10.3390/ijms21124483)
Supplement: Supplementary file 1 [file ijms-21-04483-s001.zip › Supplementary files/Supplementary Methods and Tables IJMS final revision.docx]

The circulating nucleic acid characteristics of Non-Metastatic Soft Tissue Sarcoma patients

**Supplementary Methods and Tables**

**Whole Exome Sequencing (WES):** WES of high molecular weight genomic DNA (~1µg) from normal tissue (usually buffy coat cells) or STS tissue (>50% tumour cells) was conducted by Novogene Corporation. In brief this was achieved using the Sureselect Human All Exon V6 library preparation kit followed by library preparation and Illumina short-read sequencing (depth 50-100x) using a 150bp PE kit. The fastq data files were processed using a standard bioinformatic pipeline (Broad Institute) and somatic nucleotide variants (SNV) calling using the bioinformatics tool somatic-sniper to compare STS samples with BC DNA from the same patient. Annotation of Genetic Variants (ANNOVAR) was used for variant annotation. SNVs previously associated with sarcomas or other cancers were selected to track in patient’s plasma using tNGS or ddPCR.

**Single nucleotide variant ddPCR assay primer design:** To obtain the genomic sequences surrounding each target SNV the National Centre for Biotechnology Information (NCBI) map viewer tool was used ([www.ncbi.nlm.nih.gov/mapview/](https://www.ncbi.nlm.nih.gov/mapview/)) with the hg19 assembly as a reference. Each region was checked for homology with other areas of the genome using the NCBI Blast tool (<https://blast.ncbi.nlm.nih.gov/Blast.cgi>). Following this primers for each assay were designed using the Primer3 tool (<http://primer3.ut.ee/>) with defined parameters including predicted annealing temperature (60-66^o^C), GC content (40-60%), product size (50-120) and difference in forward and reverse primer annealing temperature (<2^o^C). Potential primer pairs were analysed using the University of California, Santa Cruz’s (UCSC) genome browser’s in silico tool (<http://genome.ucsc.edu/cgi-bin/hgPcr>) to confirm specificity, followed by the Integrated DNA Technologies Oligoanalyzer tool ([www.idtdna.com/calc/analyzer](https://www.idtdna.com/calc/analyzer)) to rule out unacceptable self-dimer or primer heterodimer risk.

**Single nucleotide variant ddPCR assay Taqman hydrolysis probe design:** Two Taqman hydrolysis probes (Eurogentec, [Liège, Belgium](https://www.google.co.uk/search?dcr=0&q=Li%C3%A8ge+Belgium&stick=H4sIAAAAAAAAAOPgE-LSz9U3SM9NLjKoUuIAsVNMso20tLKTrfTzi9IT8zKrEksy8_NQOFYZqYkphaWJRSWpRcUAOLXB9kQAAAA&sa=X&ved=0ahUKEwiQ6eybqZ7aAhWGLsAKHSaGAjIQmxMImAEoATAR)) were designed for each ddPCR assay - one complementary to the mutant genotype labelled with a FAM reporter, and another complementary to the wild type genotype labelled with a HEX reporter. Black hole quenchers were attached to both probes, with LNAs as required to optimise annealing temperatures. Probes were designed with a target annealing temperature of 70^o^C, and checked using the Integrated DNA Technologies Oligoanalyzer tool for unacceptable self-dimer or primer heterodimer risk.

**Evagreen droplet digital PCR:** Evagreen ddPCR was performed using the QX200™ Droplet Digital™ PCR System as per the manufacturer’s instructions. This system consists of three instruments - the QX200 Droplet Generator, the C1000 Touch™ Thermal Cycler and the QX200 Droplet Reader. Twenty µl reaction mixes were made up with 2xQX200 ddPCR EvaGreen Supermix, Forward and Reverse primers (final concentration 250nm) and 5ng of human genomic DNA. This reaction was then mixed with 70µl of QX200 droplet generation oil for Evagreen using DG8^TM^ gaskets and cartridges, before being partitioned by the process of droplet generation using the QX200 Droplet Generator. Droplets were immediately loaded onto a 96 well plate which was sealed with a foil lid by heating to 180^o^C for 5 seconds using a PX1 PCR plate sealer. Plates were loaded onto a C1000 Touch™ Thermal Cycler where amplification was performed with the temperature ramping rate set to 2^o^C/second. Cycling conditions were 1) 95^o^C for 5 mins 2) 40 cycles of 30 seconds at 95^o^C and 1 min at 60 ^o^C 3) 5 mins at 4^o^C and 4) 5 mins at 90^o^C. Following amplification, reaction plates were loaded onto a QX200 Droplet Reader to be counted by an optical detection system. Finally raw data was uploaded into the Quantasoft^TM^ 1.7.4 software package (Bio‐Rad, CA) for analysis.

**Single Nucleotide Variant ddPCR reaction conditions:** Rare event detection ddPCR using Taqman hydrolysis probes was performed using the QX200™ Droplet Digital™ PCR System as per the manufacturer’s instructions. Twenty µl ddPCR reaction mixes were made up with 2x ddPCR™ Supermix for Probes, Forward and Reverse primers (final concentration 900nM each), mutant/wildtype probes (final concentration 250nM each) and 5ng of template DNA. Reactions were mixed with 70µl of droplet reader oil and partitioned using a QX200 Droplet Generator before being loaded onto a PCR plate and foil sealed. Reactions were processed on a C1000 Touch™ Thermal Cycler with a temperature ramping rate 2^o^C/second. The cycling conditions used for amplification were 1) 95^o^C for 10 mins 2) 40 cycles of 30 secs at 94^o^C and 60 secs at each assay’s optimum annealing temperature and 3) 98^o^C for 10mins. After amplification droplets were immediately analysed using a QX200 Droplet Reader where the number of empty, mutant and wildtype positive droplets were counted. Raw data was analysed using the Quantasoft^TM^ software package, where droplets thresholds were set in 2D prior to final data interpretation.

| **Table S1: Clinical outcome and circulating nucleic acid characteristics of STS patients** | | | | | | | | | | | |
| --- | --- | --- | --- | --- | --- | --- | --- | --- | --- | --- | --- |
| Patient | Age (yrs) / Gender | STS Subtype  (+Trojani tumour Grade) | Surgical resection margins  (Marginal / Wide) | Follow up - months | Intra-op. cfDNA level (ng/ml) | First post-op. cfDNA level (ng/ml) | STS recurrence | cfDNA level at recurrence (ng/ml) | Oncological outcome. * | Disease free survival (months) | Overall survival (months) |
| 1 | 76.1 / M | Myxofibrosarcoma (3) | Marginal (planned) | 18.8 | 9.5 | 5.5 | Metastatic | 6.5 | AWD | 18.8 | - |
| 3 | 63.1 / M | Extraskeletal Myxoid Chondrosarcoma (unknown) | Marginal (planned) | 30.1 | 15 | 7.8 | No | n/a | NED | - | - |
| 6 | 55.3 / M | Undifferentiated Pleomorphic Sarcoma (2) | 1^st^: Marginal (unplanned) 2^nd^: Wide | 9.5 | Not available | 2 | Metastatic | 3.5 | AWD | 9.5 | - |
| 9 | 62.3 / F | Leiomyosarcoma (3) | Marginal  (planned) | 1.3 | 13.8 | 3.2 | Metastatic | 3.2 | AWD | 1.3 | - |
| 10 | 59.7 / F | Synovial Sarcoma (2) | Wide | 28.9 | 9.3 | 12 | No | n/a | NED | - | - |
| 17 | 27.6 / M | Myxofibrosarcoma (1) | Wide | 17 | 15.8 | 7.3 | No | n/a | NED | - | - |
| 18 | 80.0 / F | Haemangiosarcoma (unknown) | Wide | 3.3 | Not available | 7.5 | Metastatic | 16.9 | AWD | 3.3 | - |
| 21 | 76.5 / F | Myxoid Liposarcoma (2) | Wide | 18.9 | 13.3 | 9.3 | No | n/a | NED | - | - |
| 22 | 65.4 / F | Undifferentiated Pleomorphic Sarcoma (3) | Wide | 12.6 | Not available | 9.25 | Metastatic | 16 | AWD | 12.6 | - |
| 23 | 53.2 / M | Undifferentiated Pleomorphic Sarcoma (2) | Wide | 15.6 | 2.3 | 6.3 | No | n/a | NED | - | - |
| 24 | 68.9 / M | Myxofibrosarcoma (2) | Marginal (unplanned) | 6.9 | 4.0 | 2 | Metastatic | 7.75 | DOD | 6.9 | 10.3 |
| 25 | 36.7 / F | Undifferentiated Pleomorphic Sarcoma (3) | Wide | 11.8 | Not available | 4 | unknown | 3 | Lost to FU | - | - |
| 26 | 62.8 / M | Dedifferentiated Liposarcoma (2) | Wide | 15.1 | 4.0 | 3 | No | n/a | NED | - | - |
| 27 | 67.0 / F | Undifferentiated Pleomorphic Sarcoma (2) | Wide | 11.2 | 16.1 | 10.9 | No | n/a | NED | - | - |
| 28 | 70.6 / F | Myxofibrosarcoma (2) | 1^st^: Marginal (unplanned) 2^nd^: Wide | 13.3 | 5.5 | 7 | No | n/a | NED | - | - |
| 29 | 74.0 / M | Leiomyosarcoma (3) | Marginal (planned) | 9.3 | 4.3 | 4.8 | No | n/a | NED | - | - |
| 30 | 22.2 / M | Soft Tissue Ewing’s Sarcoma (3) | Wide | 11.9 | 14.5 | 4.3 | Unknown | n/a | Lost to FU ** | - | - |
| 31 | 45.8 / M | Undifferentiated Pleomorphic Sarcoma (3) | Marginal (planned) | 4.6 | 5.8 | 30 | Metastatic | 16.75 | DOD | 4.6 | 5.3 |
| 32 | 64.0 / F | Myxofibrosarcoma (3) | 1^st^/2^nd^: Marginal (unplanned) 3^rd^: Wide | 10.2 | 4.3 | 9.5 | No | n/a | NED | - | - |
| 33 | 79.7 /M | Leiomyosarcoma (3) | Wide | 7.6 | 27.5 | 10.8 | Metastatic | 17 | AWD | 7.6 | - |
| 34 | 69.0 /M | Undifferentiated pleomorphic sarcoma (2) | 1^st^:Marginal (unplanned) 2^nd^:Wide | 8.8 | 2.5 | 15.3 | Local | n/a | NED | 30 | - |
| 35 | 87.2 / F | Myxofibrosarcoma (3) | Wide | 6.5 | 2.8 | 4.5 | No | n/a | NED | - | - |
| 36 | 74.2 / M | Dedifferentiated liposarcoma (2) | Wide | 4.9 | 35.25 | 17.3 | No | n/a | NED | - | - |
| 37 | 74.4 / F | Myxofibrosarcoma (2) | Wide | 6.5 | 6.5 | 7.8 | No | n/a | NED | - | - |
| 38 | 48.7 / F | Leiomyosarcoma (2) | Wide | 4.4 | 8.6 | 7.6 | No | n/a | NED | - | - |
| 40 | 70.3 / M | Myxofibrosarcoma (3) | 1^st^:Marginal (unplanned) | 3.8 | 24.5 | Not available | No | n/a | NED | - | - |
| 41 | 81.2 / F | Myxofibrosarcoma (3) | Wide | 4.4 | 14.3 | 19.7 | No | n/a | NED | - | - |
| 43 | 77.0 / M | Myxofibrosarcoma (2) | Wide | 2.2 | 43.6 | Not available | No | n/a | NED | - | - |
| 44 | 74.0 / M | Leiomyosarcoma (3) | Wide | 2.8 | 4.05 | Not available | No | n/a | NED | - | - |

* AWD-alive with disease; DOD-died from disease; NED-no evidence of disease.

** Patient 30 was lost to follow up precluding any comment on recurrence.

| **Table S2: IonTorrent SNV panel 2 (Sarcoma V2)** | | | | |
| --- | --- | --- | --- | --- |
| **Chromosome** | **Amplicon start point** | **Amplicon end point** | **Gene** | **Amplicon length** |
| chr13 | 48919221 | 48919308 | RB1 | 87 |
| chr13 | 48939092 | 48939157 | RB1 | 65 |
| chr13 | 48947517 | 48947590 | RB1 | 73 |
| chr13 | 48951086 | 48951167 | RB1 | 81 |
| chr13 | 48953783 | 48953872 | RB1 | 89 |
| *chr13* | *48954207* | *48954290* | *RB1* | *83* |
| *chr13* | *48955352* | *48955425* | *RB1* | *73* |
| chr13 | 48955511 | 48955594 | RB1 | 83 |
| chr13 | 49027103 | 49027187 | RB1 | 84 |
| chr13 | 49033840 | 49033932 | RB1 | 92 |
| chr13 | 49037891 | 49037961 | RB1 | 70 |
| chr13 | 49039366 | 49039457 | RB1 | 91 |
| chr13 | 49050932 | 49051028 | RB1 | 96 |
| chr17 | 7573994 | 7574075 | TP53 | 81 |
| chr17 | 7576835 | 7576924 | TP53 | 89 |
| chr17 | 7577080 | 7577173 | TP53 | 93 |
| chr17 | 7577513 | 7577609 | TP53 | 96 |
| chr17 | 7578142 | 7578234 | TP53 | 92 |
| chr17 | 7578315 | 7578409 | TP53 | 94 |
| chr17 | 7578442 | 7578529 | TP53 | 87 |
| chr17 | 7579287 | 7579381 | TP53 | 94 |
| chrX | 76778869 | 76778949 | ATRX | 80 |
| chrX | 76814193 | 76814274 | ATRX | 81 |
| chrX | 76849175 | 76849253 | ATRX | 78 |
| chrX | 76855935 | 76856021 | ATRX | 86 |
| *chrX* | *76872157* | *76872240* | *ATRX* | *83* |
| *chrX* | *76874193* | *76874277* | *ATRX* | *84* |
| chrX | 76875851 | 76875931 | ATRX | 80 |
| chrX | 76888710 | 76888791 | ATRX | 81 |
| chrX | 76889082 | 76889163 | ATRX | 81 |
| chrX | 76890067 | 76890151 | ATRX | 84 |
| *chrX* | *76890189* | *76890273* | *ATRX* | *84* |
| chrX | 76909580 | 76909666 | ATRX | 86 |
| chrX | 76931688 | 76931780 | ATRX | 92 |
| chrX | 76937042 | 76937125 | ATRX | 83 |
| chrX | 76937388 | 76937469 | ATRX | 81 |
| chrX | 76937703 | 76937790 | ATRX | 87 |
| *chrX* | *76937912* | *76938004* | *ATRX* | *92* |
| chrX | 76938321 | 76938389 | ATRX | 68 |
| chrX | 76938532 | 76938606 | ATRX | 74 |
| chrX | 76938655 | 76938734 | ATRX | 79 |
| chrX | 76939405 | 76939493 | ATRX | 88 |
| chrX | 76939541 | 76939628 | ATRX | 87 |
| chrX | 76939886 | 76939970 | ATRX | 84 |
| chrX | 76952077 | 76952162 | ATRX | 85 |

Amplicons that were excluded from the analysis due to low amplification efficiency are shown in *grey italics*.

| **Table S3: Droplet digital PCR assay design.** | | | | | |
| --- | --- | --- | --- | --- | --- |
| **Target mutation**  **(with coding strand)** | **Target strand (+/-)** | **Amplicon size (bp)** | **Assay primer sequences**  **(5'-3') (Forward/Reverse)** | **Probe sequence (5'-3') ***  **(Mutant)** | **Probe sequence (5'-3') ***  **(Wildtype)** |
| *VWDE* (7:12384078, T>C) (-) | (+) | 101 | CAATATGTATGTTCTGTTTTAGaCAA / GGGGCAACACACTCCCTACT | TTAGCCATTTGTAAATATCC[A][c]GT | TAGCCATTTGTAAATATCCA[t]GTGGAA |
| *TP53 (*17:7577022, C>T) (-) | (-) | Unknown (from Bio-Rad Laboratories, [California, United States](https://www.google.co.uk/search?dcr=0&q=Hercules+California&stick=H4sIAAAAAAAAAOPgE-LSz9U3MC4wzDVPUeIAsQsrCwu1tLKTrfTzi9IT8zKrEksy8_NQOFYZqYkphaWJRSWpRcUALCJywkQAAAA&sa=X&ved=0ahUKEwig4vmlqJ7aAhWEjKQKHbX7DSYQmxMI4AEoATAU) | | | |
| *BRIP1 (*17:59761496, C>G) (-) | (+) | 127 | TGTTAGCTAGGAGCAGAAAGTTA/ TGTTGAAAGTTGGGCTTGT | TCTTAGATGA[T][g]CAGTATTC | TCTTAGATGA[T][c]CAGTATTC |
| *PTCH1 (*9:98239884, C>A) (-) | (-) | 114 | CTCCAAGTCCCAGGGTGC/ GTTGTTGCAGCGTTAAAGGAA | CC[T][t]CAGCCACTGACAGT | CAGTCCT[g]CAGCCACTGA |
| *LPP* (3:188327063, C>A) (+) | (-) | 98 | CCAACCCCCTCTAACAGCAA / TGTTCCGATTGGAGCCACAG | TACATTGAA[A][a]CACAGCCTGCAC | TACATTGAAA[c]CACAGCCTGCAC |
| *FLT4 (*5:180046092, G>A) (-) | (-) | 91 | GAAGTTGGAGAGGTTGCC/ GGTTCCGAACGCACG | C[A][t][G][A]TCACCATGAGG | C[A][c][G]ATCACCATGAGG |
| *DACH1 (*13: 72053389, A>C) (-) | (+) | 123 | GGCTGTTGAAAGTTGCCAT / ACTGCTTCTCAAGTGTTTCCC | CCAACTGG[A][c]AAAACTG | CCAACTGG[A][a]AAAACTG |
| *EPHB6 (*7:142563798, G>A) (+) | (+) | 73 | GCAAGGCTCAGCACTCATG / ATTGAAGAGCAGGTCCCCTC | ACCC[C][t]CAGCTCC | ACCC[C][c]CAGCTCC |
| *MMS22L* (6:97634424, C>T) (-) | (+) | 126 | GTACAGTCTTCATTATCGGCTA / CGCAAGTTGTGAGAAAGG | AAGAG[T][t]AGAGAATGTCAC | AAGAGT[c]AGAGAATGTCAC |
| *ITIH2 (*10:7769692, C>T) (+) | (-) | 143 | ATGGGATTTTCTGCGTGTG / GAGTTGGGGTCTAACAGTCC | CACTCCT[A][t][G]GGCAATCTTC | CACTCCT[A][c]GGGCAATCTTC |
| *KDM5B* (1:202777369, C>T) (-) | (-) | 140 | GTGGATGAAAGCGAAGGGG / CCACCACACTGCACCCAG | AACTCGCCCAGC[a]G | AACTCGCCCAGC[g]G |
| *PTPRB* (12:70970320, C>T) (-) | (+) | 86 | CGGTGAACAATTCCGGTCGT / ACTGAACCACCTTGCCGTCA | ATAACTATGAG[G]T[A][A][t][A]T | TATGAGGTA[A][c][A]TTGTCTC |

***** Taqman hydrolysis probe bases enclosed in square brackets represent locked nucleic acids (LNAs). Small case characters in the probes represent the sites of the target mutations.

| **Table S4: IonTorrent Sarcoma SNV Ampliseq pools (Sarcoma V345)** | | | | | | |
| --- | --- | --- | --- | --- | --- | --- |
| **Patient** | **Chr.** | **Amplicon start point** | **Amplicon end point** | **Gene** | **Amplicon length** | **tNGS Ampliseq pool** |
| 31 | 6 | 51695706 | 51695749 | PKHD1 | 43 | V3 |
| 31 | 21 | 41550914 | 41550951 | DSCAM | 37 | V3 |
| 31 | 22 | 30035146 | 30035192 | NF2 | 46 | V3 |
| 34 | 5 | 71492808 | 71492858 | MAP1B | 50 | V3 |
| 34 | 9 | 95373574 | 95373608 | CENPP | 34 | V3 |
| 34 | X | 101152970 | 101153014 | ZMAT1 | 44 | V3 |
| *35* | *2* | *212652717* | *212652772* | *ERBB4* | *55* | V3 |
| *35* | *3* | *48502021* | *48502071* | *ATRIP* | *50* | V3 |
| 35 | 10 | 49400720 | 49400768 | FRMPD2 | 48 | V3 |
| 35 | 13 | 29068931 | 29068969 | FLT1 | 38 | V3 |
| *35* | *19* | *51329049* | *51329104* | *KLK15* | *55* | V3 |
| *35* | *19* | *15082658* | *15082712* | *SLC1A6* | *54* | V3 |
| 36 | 2 | 85852647 | 85852693 | USP39 | 46 | V4 |
| 36 | 2 | 227906861 | 227906917 | COL4A4 | 56 | V4 |
| 36 | 3 | 46062647 | 46062701 | XCR1 | 54 | V4 |
| *36* | *6* | *142759370* | *142759419* | *ADGRG6* | *49* | V4 |
| 36 | 10 | 72492003 | 72492056 | ADAMTS14 | 53 | V4 |
| 37 | 2 | 166187891 | 166187940 | SCN2A | 49 | V3 |
| *37* | *12* | *66786145* | *66786202* | *GRIP1* | *57* | V3 |
| *37* | *14* | *95658999* | *95659036* | *CLMN* | *37* | V3 |
| 38 | 2 | 27549519 | 27549572 | GTF3C2 | 53 | V4 |
| *38* | *3* | *52582202* | *52582245* | *PBRM1* | *43* | V4 |
| 38 | 4 | 187455553 | 187455586 | MTNR1A | 33 | V4 |
| 38 | 13 | 49027106 | 49027145 | RB1* | 39 | V4 |
| 40 | 4 | 115544314 | 115544361 | UGT8 | 47 | V4 |
| *40* | *4* | *26484826* | *26484868* | *CCKAR* | *42* | V4 |
| 40 | 8 | 113702186 | 113702232 | CSMD3 | 46 | V4 |
| 40 | 11 | 134177007 | 134177043 | GLB1L3 | 36 | V4 |
| 40 | 15 | 52497020 | 52497075 | MYO5C | 55 | V4 |
| 40 | 15 | 65449198 | 65449235 | CLPX | 37 | V4 |
| *41* | *2* | *75101482* | *75101513* | *HK2* | *31* | V5 |
| *41* | *5* | *112178496* | *112178542* | *APC* | *46* | V5 |
| 41 | 5 | 45262553 | 45262597 | HCN1 | 44 | V5 |
| 41 | 7 | 91737896 | 91737948 | AKAP9 | 52 | V5 |
| 41 | 22 | 32482238 | 32482285 | SLC5A1 | 47 | V5 |
| 43 | 3 | 183681182 | 183681232 | ABCC5 | 50 | V5 |
| 43 | 5 | 14367021 | 14367074 | TRIO | 53 | V5 |
| 43 | 5 | 137895629 | 137895678 | HSPA9 | 49 | V5 |
| 43 | 8 | 57078902 | 57078957 | PLAG1 | 55 | V5 |
| 43 | 11 | 126075430 | 126075463 | RPUSD4 | 33 | V5 |
| *43* | *12* | *107393391* | *107393442* | *CRY1* | *51* | V5 |
| *43* | *17* | *7230122* | *7230181* | *NEURL4* | *59* | V5 |
| *43* | *19* | *12821432* | *12821490* | *TNPO2* | *58* | V5 |
| *44* | *13* | *103288612* | *103288659* | *TPP2* | *47* | V5 |
| 44 | 17 | 7577080 | 7577127 | TP53 | 47 | V5 |
| *44* | *19* | *6697434* | *6697491* | *C3* | *57* | V5 |

Amplicons that were excluded from the analysis due to low amplification efficiency, low level variant detection in all samples (>0.1%), or allele drop out are shown in *grey italics*.

* The RB1 amplicon (patient 38) in the Ion Torrent Ampliseq panel showed poor amplification. It was replaced by an alternative amplicon developed in house.

| **Supplementary Table 5. Analysis of ctDNA using the IonTorrent Sarcoma V345 panel** | | | | | | | | **cfDNA** | | **Tumour DNA** | | **BC DNA** | |
| --- | --- | --- | --- | --- | --- | --- | --- | --- | --- | --- | --- | --- | --- |
| **Pt31,34,35,37- tNGS pool V3** | **Gene** | **SNV** | **Variant Frequency in Tumour WES (%)** | **Tumour %** | **Comments** | **Chr** | **Coordinate** | **Ion Torrent read depth** | **SNV detected %** | **Ion Torrent read depth** | **SNV detected %** | **Ion Torrent read depth** | **SNV detected %** |
| **Pt 31**  **( T23)** | NF2 | C>T | 60 | 75 | COSMIC ID: COSM6848123 | 22 | 30035181 | 3992 | 0.05% | 3214 | yes 27% | - | - |
|  | PKHD1 | C>T | 24 | 75 | mutated in >1% of sarcomas | 6 | 51695719 | 22089 | 0.02% | 26431 | yes 20% | - | - |
|  | DSCAM | G>A | 22 | 75 | mutated in >1% of sarcomas | 21 | 41550926 | 15107 | 0.05% | 25448 | yes 16% | - | - |
| **Pt 34**  **(T24)** | ZMAT1 | C>T | 44 | 70 | mutated in >1% of sarcomas | X | 101153003 | 1515 | 0.00% | 50 | yes 46% | - | - |
|  | CENPP | G>T | 27 | 70 | * cancer associated - mouse | 9 | 95373595 | 7096 | 0.00% | 485 | yes 14% | - | - |
|  | MAP1B | C>T | 20 | 70 | * cancer associated - mouse | 5 | 71492844 | 3916 | 0.00% | 215 | yes 17% | - | - |
| **PT35**  **(T25)** | FRMPD2 | C>T | 46 | 90 |  | 10 | 49400730 | 8159 | 0.06% | 5355 | yes 20% | 897/8098 | 0.20% |
|  | FLT1 | G>A | 45 | 90 | * cancer associated - mouse | 13 | 29068938 | 10336 | 0.07% | 3780 | yes 36% | 907/8203 | 0.07% |
| **PT37 (T26)** | SCN2A | T>C | 20 | 80 | mutated in >1% of sarcomas | 2 | 166187927 | 578 ** | 0.17% | 362 | yes 24% | - | **-** |
| **PT36,38.40 - tNGS pool V4** |  |  |  |  |  |  |  |  |  |  |  |  |  |
| **Pt 36**  **(T27)** | XCR1 | C>T | 44 | 80 | COSMIC ID: COSM4166892 | 3 | 46062685 | 832/14809 | 0.08% | 7665 | yes 41% | - | - |
|  | USP39 | T>G | 44 | 80 | * cancer associated - mouse | 2 | 85852681 | 510/7705 | 0.00% | 2938 | yes 49% | - | - |
|  | ADAMTS14 | G>A | 44 | 80 |  | 10 | 72492032 | 1852/26794 | 0.02% | 12150 | yes 42% | - | - |
|  | COL4A4 | G>A | 40 | 80 | mutated in >1% of sarcomas | 2 | 227906875 | 1096/17230 | 0.03% | 6185 | yes 46% | - | - |
| **Pt 38**  **(T28)** | RB1 | G>C | 76 | 65 | Cosmic known cancer gene | 13 | 49027128 | 186458 | 0.001% | 60874 | yes 52% | 59456 | 0.002% |
|  | MTNR1A | C>T | 44 | 65 | * cancer associated - mouse | 4 | 187455565 | 1016/2950 | 0.00% | 11156 | yes 37% | - | - |
|  | GTF3C2 | T>C | 40 | 65 | mutated in >1% of all cancers | 2 | 27549568 | 2722/7339 | 0.07% | 21470 | yes 34% | - | - |
| **Pt 40**  **(T30)** | MYO5C | A>T | 84 | 95 | * cancer associated - mouse | 15 | 52497065 | 2052 | 0.00% | 2766 | yes 85% | 897/2698 | 0.07% |
|  | GLB1L3 | G>T | 64 | 95 | mutated in >1% of sarcomas | 11 | 134177026 | 3451 | 0.00% | 827 | yes 58% | 470/1004 | 0.00% |
|  | CSMD3 | A>G | 63 | 95 | COSMIC ID: COSM1095155 | 8 | 113702211 | 866 | *** 0.35% | 1834 | yes 44% | 351/770 | 0.13% |
|  | CLPX | T>G | 42 | 95 | * cancer associated - mouse | 15 | 65449223 | 8486 | 0% | 15603 | yes 38% | 1932/4650 | 0.06% |
|  | UGT8 | G>A | 50 | 95 | COSMIC ID: COSM201859 | 4 | 115544340 | 1871 | 0.11% | 1932 | yes 21% | 314/670 | 0.15% |
| **Pt41,43,44- tNGS pool V5** |  |  |  |  |  |  |  |  |  |  |  |  |  |
| **PT41**  **(T31)** | SLC5A1 | G>T | 21 | 95 | * cancer associated - mouse | 22 | 32482259 | 3158 | 0.00% | 1646 | yes 13% | - | - |
|  | AKAP9 | G>C | 21 | 95 | Cosmic putative cancer gene | 7 | 91737924 | 2245 | 0.04% | 1629 | yes 28% | - | - |
|  | HCN1 | C>T | 22 | 95 | * cancer associated - mouse | 5 | 45262589 | 3258 | 0.00% | 1967 | yes 25% | - | - |
| **Pt 43**  **(T32)** | ABCC5 | G>A | 67 | 75 | * cancer associated - mouse | 3 | 183681223 | 3939 | yes 1.19% | 2889 | yes 58% | 6195 | 0.03% |
|  | RPUSD4 | G>A | 56 | 75 | * cancer associated - mouse | 11 | 126075438 | 2748 | strand bias 2263+/529-; 0.29% | 14657 | yes 43% | 5118 | 0.18% |
|  | TRIO | C>T | 49 | 75 | * cancer associated - mouse | 5 | 14367062 | 5670 | yes 0.65% | 17322 | yes 41% | 9762 | 0.05% |
|  | PLAG1 | C>A | 48 | 75 | Cosmic known cancer gene | 8 | 57078921 | 2259 | yes 0.93% | 5230 | yes 45% | 4903 | 0.00% |
|  | HSPA9 | C>T | 41 | 75 | * cancer associated - mouse | 5 | 137895674 | 5936 | yes 0.83% | 25278 | yes 46% | 13449 | 0.07% |
| **Pt 44 (T33)** | TP53 | G>C | 38 | 90 | mutated in >1% of sarcomas | 17 | 7577111 | 4891 | 0.04% | 9457 | yes 35% | 21836 | 0.02% |

SNV alleles detected at <0.1% were deemed to be background noise and disregarded

SNV alleles detected at >0.5% in cfDNA were taken as positive evidence of ctDNA

* Mouse insertional mutagenesis experiments support the designation of gene as a cancer causing gene

** Read depth low in cfDNA and tumour samples from Pt37 but averaged 988 reads across the other samples assayed using the Ampliseq pool v4

*** This amplicon showed unexpected strand bias in the cfDNA (61+/805-) that was not seen in tumour or BC DNA); the variant allele was detected at <0.5% in the cfDNA below the threshold set for evidence of ctDNA.

**Table S6: Patient enrolment criteria.**

| Inclusion Criteria | Exclusion Criteria |
| --- | --- |
| Patients with non-metastatic biopsy proven STS | Patients presenting with local or distant STS recurrence |
| Patients undergoing attempted curative surgical resection (+/adjuvant therapy) | Patients with retroperitoneal STS |
| Patient management and follow up planned under the remit of the East Midlands Sarcoma Service | Patients unable to provide informed consent |
|  | Patients aged under 18 |

Enrolled patients were removed from the project when they 1) developed disease recurrence, 2) were lost to follow up or 3) voluntarily withdrew.
